# 

**Published:** 1877-06

**Authors:** 


### Contents of June Number.

### ORIGINAL.

ART. I.—On Albumen in the treatment of Pulmonary Consumption, by E. L. Shurly, M. D.,.... ............ .................... 283
ART. II.—The Hip-Joint. Paper read before Buffalo Medical Association, May 1st, 1877, by E. R. Barnes, M. D.,................ 292
ART. III.—Buffalo Medical Club, by E. N. Brush, M. D,,............ 299

### MISCELLANEOUS.

The Abuse and Use of Bromides,........ ........................ 303
An Expose of Opium "Antidotes,"................................ 308
Proceedings of Norfolk District Medical Society,............ ...... 310
Relief of Pain by the outward application of Hydrate of Chloral,...... 313
Action of Gelseminum Senpervirens,................ ............. 314
Bromide of Potassium as a Caustic, ............................ 314

### EDITORIAL.

Valuable New Books,................ .......... ........ ........ 315
Meeting of the New York State Medical Society,.................... 315
Death of Dr. Charles Winne,............... ........... ...... 315
Meeting of the New York State Medical Society,.................... 317
Convention of the Kentucky State Medical Society,............... .... 317
American Gynæcological Society,................................ 318
American Medical Colleges,...... ......................... ..... 318

BOOKS REVIEWED:

A Practical Treatise on Diseases of the Eye, by R. B. Carter, F. R. C. S.,........................... ........... ........ 319 319
Analysis of the Urine, by Geo. B. Fowler, M. D.,............ 320
Ziemssen's Cyclopœdia, Vol. VI,......................... 320
Theory and Practice of Medicine, by J. S. Bristowe, M. D., Lonel, F. R. C. P.,.............................. 322

Books and Pamphlets Received,.................................. 322

